# CXCL12-CXCR4-Mediated Chemotaxis Supports Accumulation of Mucosal-Associated Invariant T Cells Into the Liver of Patients With PBC

**DOI:** 10.3389/fimmu.2021.578548

**Published:** 2021-03-19

**Authors:** Zhilei Chen, Suying Liu, Chengmei He, Jinlei Sun, Li Wang, Hua Chen, Fengchun Zhang

**Affiliations:** ^1^Department of Rheumatology and Clinical Immunology, Peking Union Medical College Hospital, Chinese Academy of Medical Sciences & Peking Union Medical College, Beijing, China; ^2^Key Laboratory of Rheumatology and Clinical Immunology, Ministry of Education, Beijing, China

**Keywords:** primary biliary cholangitis, mucosal-associated invariant T cells, chemotaxis, CXCR4, CXCL12

## Abstract

**Objectives:** To explore the potential role of CD3^+^CD8^+^CD161^high^ TCRVα7.2^+^ mucosal-associated invariant T (MAIT) cells in the pathogenesis of primary biliary cholangitis (PBC).

**Methods:** We enrolled 55 patients with PBC, 69 healthy controls (HCs), and 8 patients with hepatic hemangioma. Circulating MAIT cells and their chemokine receptor profiles and cytokine production were quantified using flow cytometry. Liver-resident MAIT cells were examined by immunofluorescence staining. CXCL12-mediated chemotaxis of MAIT cells was measured using a transwell migration assay. Plasma interleukin (IL)-18 was measured using ELISA, and cytokine production in IL-18-stimulated MAIT cells was detected using flow cytometry.

**Result:** Peripheral MAIT cells were found to be significantly lower in patients with PBC (3.0 ± 3.2% vs. 9.4 ± 8.0%, *p* < 0.01) and negatively correlated with alkaline phosphatase (ALP) levels (*r* = −0.3209, *p* < 0.05). Liver immunofluorescence staining suggested that MAIT cells might accumulate in PBC liver. MAIT cells from patients with PBC expressed higher levels of CXCR4 (84.8 ± 18.0% vs. 58.7 ± 11.4%, *p* < 0.01), and the expression of CXCL12 was higher in PBC liver. CXCL12 promoted MAIT cell chemotaxis (70.4 ± 6.8% vs. 52.2 ± 3.5%, *p* < 0.01), which was attenuated by CXCR4 antagonist. MAIT cells from PBC produced significantly more interferon-γ (IFN-γ) (88.3 ± 4.2% vs. 64.2 ± 10.1%, *p* < 0.01), tumor necrosis factor-α (TNF-α) (93.0 ± 1.1% vs. 80.1 ± 5.3%, *p* < 0.01), Granzyme B (89.3 ± 3.3% vs. 72.1 ± 7.0%, *p* < 0.01), and perforin (46.8 ± 6.6% vs. 34.8 ± 7.7%, *p* < 0.05). MAIT cells from PBC expressed higher levels of IL18-Rα (83.8 ± 10.2% vs. 58.3 ± 8.7%, *p* < 0.01). Plasma IL-18 was more abundant in patients with PBC (286.8 ± 75.7 pg/ml vs. 132.9 ± 78.1 pg/ml, *p* < 0.01). IL-18 promoted IFN-γ production in MAIT cells (74.9 ± 6.6% vs. 54.7 ± 6.7%, *p* < 0.01), which was partially attenuated by blocking IL-18R (68.6 ± 8.3% vs. 43.5 ± 4.2%, *p* < 0.01).

**Conclusion:** Mucosal-associated invariant T cells from patients with PBC accumulated in the liver *via* CXCL12-CXCR4-mediated chemotaxis, produced pro-inflammatory cytokines, and contributed to portal inflammation, which was potentially mediated by elevated IL-18. Targeting MAIT cells might be a therapeutic approach for PBC.

## Introduction

Primary biliary cholangitis (PBC) is a chronic autoimmune cholestatic liver disease characterized by non-suppurative inflammation and immune-mediated destruction of small intrahepatic bile ducts, portal tract fibrosis, cirrhosis, and circulating anti-mitochondrial antibodies (1). PBC also presents systemic involvements beyond cholangitis, including interstitial lung disease, (2) pulmonary hypertension (3), and cardiomyopathy (4). The therapeutic approaches for PBC remain limited: ursodeoxycholic acid (UDCA) and obeticholic acid (Ocaliva) are the only FDA-approved agents, with ~60% biochemical response to UDCA (1, 5). Fibrates, methotrexate, colchicine, azathioprine, and liver transplant are unapproved alternatives (6, 7). Thus, investigation of the pathogenesis of PBC and potential therapeutic targets is urgently needed.

Mucosal-associated invariant T (MAIT) cells are a subset of unconventional T cells expressed as evolutionarily conserved invariant T-cell antigen receptors (TCRs), including semi-invariant α-chain Vα7.2-Jα33-20-12 in human and Vα19-Jα33 in mice (8). MAIT cells present in the peripheral blood and tissues in the gut and the liver (9, 10) actively produce interferon-γ (IFN-γ), interleukin (IL)-17, Granzyme B, and perforin in response to viral and bacterial challenges and show pro-inflammatory and cytotoxic phenotypes (11). Moreover, MAIT cells are implicated in liver diseases, including autoimmune liver disease and alcoholic or non-alcoholic hepatitis (12–14).

Mucosal-associated invariant T cells have been studied in PBC. Setsu et al. (15) report that MAIT cells are persistently activated and exhausted in patients with PBC and are lower in the peripheral blood and the liver. Jiang et al. (16) found reduced circulating MAIT cells but abundant liver-resident MAIT cells in patients with PBC, which is activated by IL-7 produced by cholic acid-stimulated hepatocytes. These studies have suggested that MAIT cells are potentially implicated in PBC. However, whether, and how, MAIT cells aggregate in the liver of patients with PBC remain unknown.

To address this issue, we analyzed the population and immunophenotype of peripheral MAIT cells from patients with PBC. We further analyzed the liver-resident MAIT cells in patients with PBC. Finally, we explored the underlying mechanism of liver accumulation and hyperactivation of MAIT cells from patients with PBC.

## Materials and Methods

### Patients and Healthy Controls

We enrolled 55 patients with PBC who fulfilled the consensus criteria of PBC and were admitted to the Peking Union Medical College Hospital (PUMCH) from July 2017 to September 2019 (Table 1) (17). Substantial higher alkaline phosphatase (ALP), gamma-glutamyl transferase, and total bile acid were found in patients with PBC. We also enrolled 69 age- and sex-matched healthy volunteers as healthy controls (HCs). Additionally, eight patients with hepatic hemangioma who underwent surgical resection were enrolled as controls. This study was approved by the institutional review board of PUMCH. Written informed consent was obtained from all participants.

**Table 1:** Demographic and clinical characteristics of patients with PBC and healthy controls (HCs).

**Parameter**	**PBC (*n =* 55)**	**HCs (*n =* 69)**	***p-*value**
Age—year	52.55 ± 11.88	49.45 ± 8.88	0.10
Female—sex	55 (100%)	69 (100%)	1.00
ALP—U/L	112 (84–202)	64 (56–79.5)	<0.001
GGT—U/L	53 (27–107)	16 (13.5–27.5)	<0.001
ALT—U/L	31 (20–53)	16 (12–19)	<0.001
AST—U/L	37 (25–47)	18 (15–22)	<0.001
TP—g/l	76.73 ± 5.60	73.36 ± 4.35	0.03
ALB—g/l	42.09 ± 3.85	45.19 ± 3.03	0.35
TBA—μmol/l	6.20 (2.90 ± 20.40)	3.20 (1.95 ± 5.25)	<0.001
TBIL—μmol/l	13.51 ± 3.77	10.80 ± 4.78	0.18
DBIL—μmol/l	4.83 ± 2.08	3.84 ± 1.31	0.002
TC—mmol/l	5.05 ± 1.16	4.80 ± 0.93	0.49
TG—mmol/l	1.27 ± 0.72	1.42 ± 1.07	0.17
HDL—mmol/l	2.15 (1.47–3.23)	1.28 (1.15–1.55)	<0.001
LDL—mmol/l	1.72 (1.39–2.81)	2.6 (2.30–3.19)	<0.001
Cr—μmol/l	61.13 ± 13.09	61.04 ± 10.77	0.25
BUN—μmol/l	4.75 ± 1.30	4.45 ± 1.00	<0.01
UA—mmol/l	295.8 ± 68.19	282.8 ± 74.66	0.40
IgG—g/l	15.42 (13.45–18.1)	NA	NA
IgA—g/l	3.10 (2.37–4.24)	NA	NA
IgM—g/l	2.40 (1.51–3.5)	NA	NA
ANA—%	54 (98.19%)	NA	NA
AMA—%	51 (92.73%)	NA	NA
Anti-GP210 (*n =* 15)	8 (53.33%)	NA	NA
Anti-SP100 (*n =* 15)	10 (66.67%)	NA	NA

*Values were presented as mean ± SD, median (interquartile range), or number (percentage). The data were analyzed using Kolmogorov–Smirnov test, Student's t-test and Mann–Whitney U test*.

*ALB, albumin; ALP, alkaline phosphatase; ALT, alanine aminotransferase; AMA, antimitochondrial antibody; ANA, antinuclear antibody; AST, aspartate transaminase; BUN, blood urea nitrogen; Cr, Creatinine; DBIL, direct bilirubin; GGT, gamma-glutamyl transferase; GP210, glycoprotein-210; HDL, high-density lipoprotein; IgA, immunoglobulin A; IgG, immunoglobulin G; IgM, immunoglobulin M; LDL, low-density lipoprotein; TBA, total bile acid; TBIL, total bilirubin; TC, total cholesterol; TG, total triglycerides; TP, total protein; UA, uric acid*.

### Flow Cytometry

Peripheral blood mononuclear cells (PBMCs) were isolated with Ficoll gradient and maintained in RPMI-1640 medium supplemented with 10% fetal bovine serum (FBS) (Gibco, MD, USA) at 37°C. MAIT cells were stimulated with phorbol myristate acetate (PMA, 5 ng/ml) (Sigma-Aldrich, St. Louis, MO, USA), ionomycin (1 μg/ml) (Sigma-Aldrich, St. Louis, MO, USA), and brefeldin A (Biolegend, San Diego, CA, USA) for 2.5 h or anti-CD3 (5 μg/ml) (BD Biosciences, San Jose, USA) and anti-CD28 (5 μg/ml) (BD Biosciences, San Jose, USA) for 72 h. Intracellular staining was performed as previously described (18). Cell proliferation and apoptosis were detected using CFSE dilution (BD Biosciences, San Jose, CA, USA) and Annexin V Apoptosis Detection Kit (BD Biosciences, San Jose, CA, USA). Data were acquired on a BD Aria II Cytometry (BD Biosciences, San Jose, CA, USA) and were analyzed using FlowJo software (Treestar, OR, USA).

### Antibodies

Anti-CD3 (UCHT1), anti-CD8a (SK1), anti-TCRVα7.2 (3C10), anti-CD161 (DX12), anti-CD69 (FN50), anti-CD38 (HIT2), anti-IL17A (BL168), anti-IFN-γ (4S.B3), anti-CXCR4 (12G5), anti-TNF-α (MAb11), anti-Granzyme B (GB11), anti-perforin (B-D48), anti-IL-18Rα (H44), anti-CCR5 (J418F1), anti-CCR6 (G034E3), anti-CCR10 (6588-5), anti-CX3CR1 (2A9-1), anti-CXCR1 (8F1/CXCR1), anti-CXCR3 (G025H7), anti-CXCR5 (J252D4), and anti-CXCR6 (K041E5) antibodies were purchased from BD Biosciences (San Jose, CA, USA) and BioLegend (San Diego, CA,USA).

## Cytokine Measurement

IL-18 was measured using the IL-18/IL-18 BPa Complex DuoSet ELISA (R&D Systems, MN, USA) according to the instructions of the manufacturer. All tests were performed in duplicate.

### Immunohistochemistry and Immunofluorescence

Liver tissues fixed with paraformaldehyde (PFA) were stained with anti-CXCL12 (R&D Systems, MN, USA) antibody. In brief, the tissue sections were deparaffinized, rehydrated, and treated with an EDTA buffer antigen retrieval solution for 26 min. After blocking endogenous peroxidase with 3% H_2_O_2_ and 3% BSA at room temperature for 30 min, slides were incubated with a mouse anti-CXCL12 antibody (R&D Systems, MN, USA) overnight at 4°C. The slides were washed and covered with HRP-conjugated anti-mouse antibodies (Servicebio, Wuhan, China) at room temperature for 50 min. The slides were developed with diaminiobenzidine (DAB), counterstained in the nucleus with hematoxylin, washed, dehydrated in series-gradient ethanol, washed, and mounted on a microscope. The density of CXCL12^+^ cells (cells/mm^2^) was quantified using ImageJ [National Institutes of Health (NIH)].

Immunofluorescence staining was performed on paraffin-embedded liver tissues using the following antibodies: rabbit anti-human CD161 (ab197979, Abcam, MA, USA), CY3 goat anti-rabbit (Servicebio, Wuhan, China), and FITC mouse anti-human TCRVα7.2 antibodies. After staining with DAPI, images were acquired using fluorescent microscopy (NIKON Eclipse Ti). The density of CD161^+^TCRVα 7.2^+^ cells (cells/mm^2^) was quantified using Image J (NIH).

## Chemotaxis Assay

CD8^+^ T cells from patients with PBC were starved in RPMI-1640 medium containing 1% FBS overnight. The cells were seeded in the upper transwell chamber (Corning, NY, USA), and media containing 0, 10, 30, or 50 ng/ml CXCL12 (Peprotech, NJ, USA) were added to the lower chamber for 1.5 h. Cells in both chambers were harvested and mixed with 123 count eBeads (Thermo Fisher Scientific, MA, USA); stained with fluorochrome-conjugated anti-CD3, anti-CD8a, anti-TCRVα7.2, and anti-CD161 antibodies; and processed with a flow cytometer. Chemotaxis rate was calculated as the percentage of MAIT cells in the lower chamber of the total MAIT cells. For blocking CXCR4, the cells were pre-treated with AMD3100 (1 μg/ml) (TOCRIS, Abingdon, UK) for 30 min before loading onto the upper chamber.

## IL-18 Stimulation Assay

Peripheral blood mononuclear cells from patients with PBC were stimulated with plate-bound anti-CD3 (5 μg/ml), anti-CD28 (5 μg/ml), and IL-18 (100 ng/ml), (BioLegend, San Diego, CA,USA) at 37°C for 48 h. Cells were harvested, and the production of IFN-γ was measured using a flow cytometer. For blocking IL-18 R, the cells were pre-treated with anti-IL-18Rα/IL-1R5 (1 μg/ml) (R&D Systems, MN, USA) antibody for 30 min.

### Statistical Analysis

The data were summarized as the mean ± SD, median (interquartile range), or number (percentage). For normal distribution data, the Student's *t*-test was used to compare the differences between the two groups. A paired *t*-test was used to compare the differences between the paired samples at different conditions. Non-normal distribution data were analyzed using the Kolmogorov–Smirnov test, ANOVA, and the Mann–Whitney U test. Correlations were calculated using Spearman's correlation analysis. A two-sided *p* < 0.05 was considered statistically significant. The data were analyzed using the SPSS V.17.0 software (IBM, NY, USA).

## Results

### Lower Peripheral MAIT Cells From Patients With PBC

**Figure 1:**
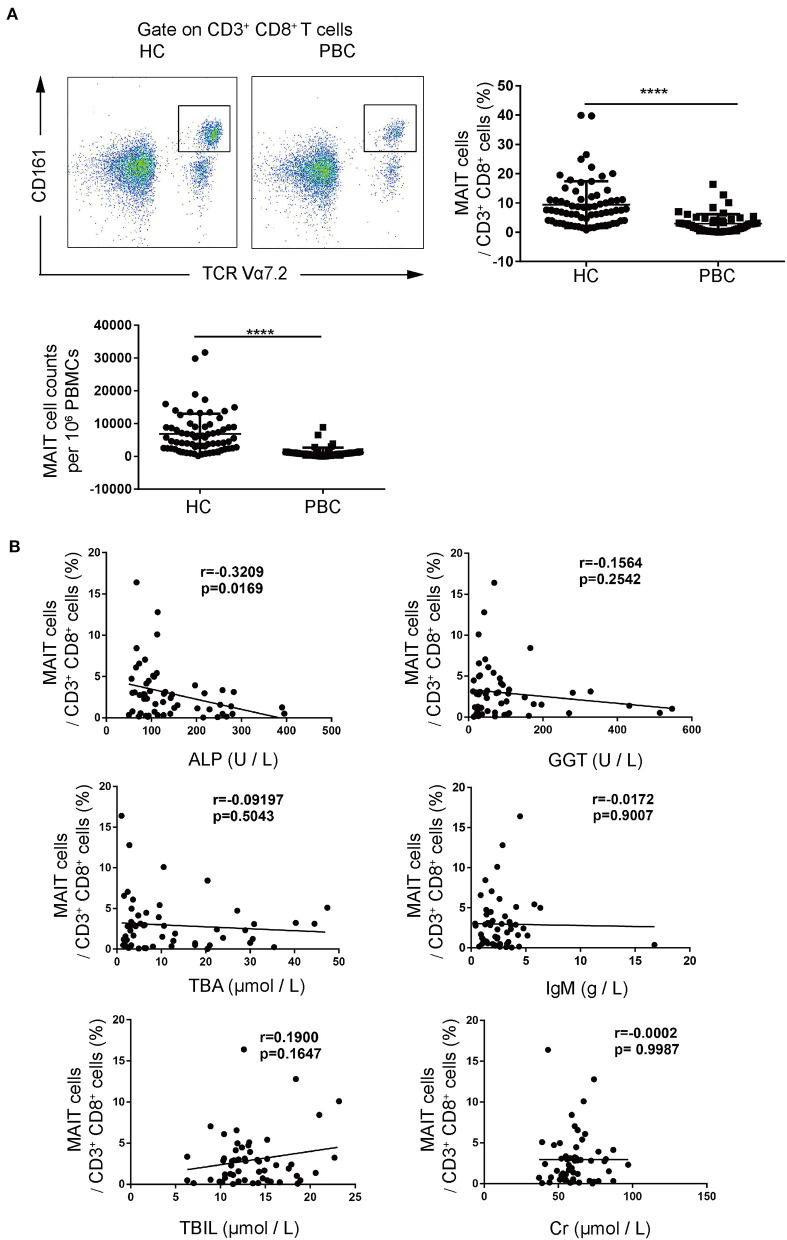
Peripheral mucosal-associated invariant T cells (MAIT) cells from patients with primary biliary cholangitis (PBC) decreased significantly. **(A)** Representative plots of fluorescence-activated cell (FACS) (left), summary frequency (right), and absolute numbers (bottom) of peripheral CD3^+^CD8^+^CD161^high^TCRVα7.2^+^MAIT cells from patients with PBC (*n* = 55) and healthy controls (HCs) (*n* = 69). **(B)** Correlation of peripheral MAIT cell population with laboratory parameters in patients with PBC (*n* = 55). Data were expressed as mean ± SD. The value of ****p < 0.0001 by the Student's *t-*test. Correlations were calculated using the Spearman's correlation analysis.

We first examined CD3^+^CD8^+^CD161^high^TCRVα7.2^+^ MAIT cells in PBMCs from patients with PBC and from HCs. We found that the frequencies of MAIT cells in CD3^+^CD8^+^T cells from patients with PBC were significantly lower than those from HCs (3.0 ± 3.2% vs. 9.4 ± 8.0%, *p* < 0.01, Figure 1A). Consistently, the number of circulating MAIT cells in PBMCs from patients with PBC was lower than those from HCs (1,186 ± 1,491 vs. 6,866 ± 6,154 cells per 10^6^ PBMCs, *p* < 0.01, Figure 1A). Furthermore, circulating MAIT cells from patients with PBC negatively correlated with ALP (*r* = −0.3209, *p* < 0.05, Figure 1B), a PBC disease activity maker (19). No significant correlation was observed between γ-glutamyltransferase (GGT), total bile acid (TBA), total bilirubin (TBIL), creatinine (Cr) (Figure 1B), direct bilirubin (DBIL), alanine aminotransferase (ALT), total cholesterol (TC), total triglycerides (TG), uric acid (UA), and immunoglobulin G (Supplementary Figure 1). Collectively, MAIT cells decreased in the peripheral blood of patients with PBC and inversely correlated with the disease activity of PBC, suggesting that MAIT cells might play a role in the pathogenesis of PBC.

### MAIT Cells From Patients With PBC Show a Higher Potential of Inflammation

**Figure 2:**
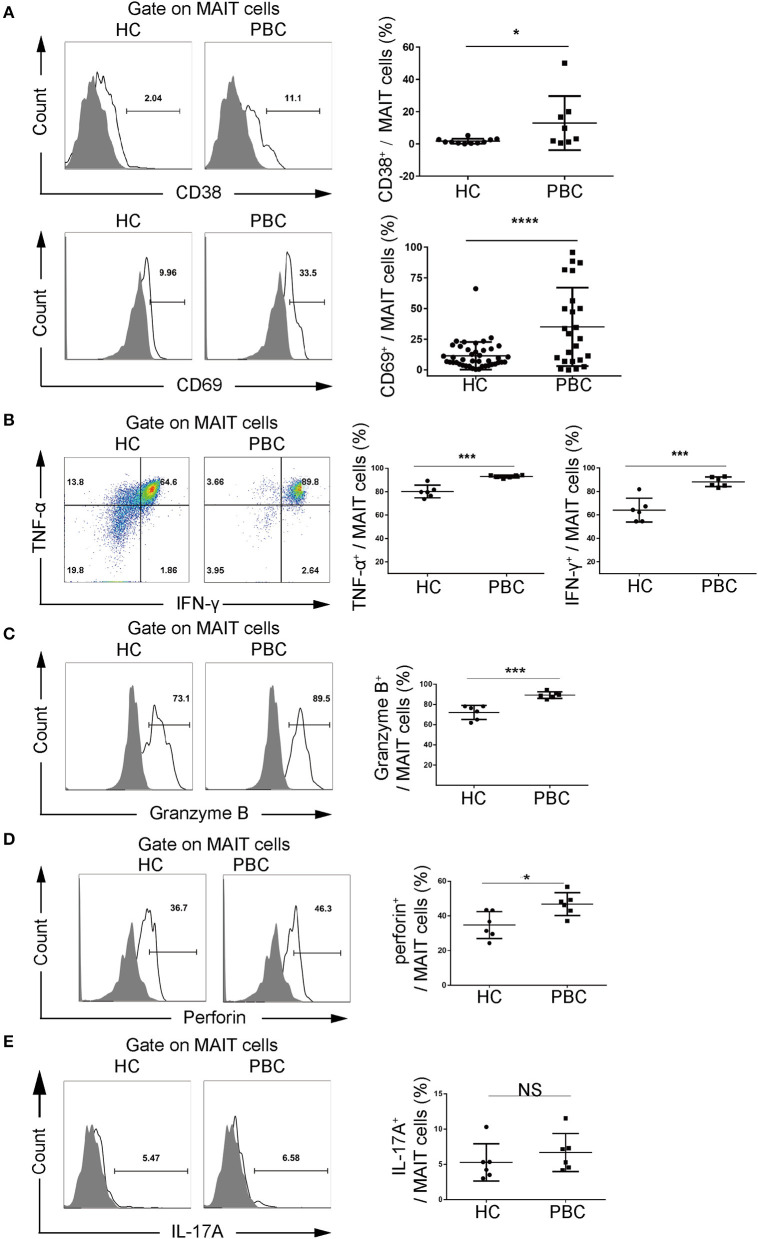
Activation potential of MAIT cells from patients with PBC. Peripheral blood mononuclear cells (PBMCs) were stimulated with phorbol myristate acetate (PMA) and ionomycin or anti-CD3 and anti-CD28 antibodies. Representative FACS plots (left) and summary graphs (right) of **(A)** CD38 (upper, PBC = 8, HC = 11) and CD69 (bottom, PBC = 24, HC = 42) expression on MAIT cells from patients with PBC and from HCs. **(B)** Interferon-γ (IFN-γ) and tumor necrosis factor-α (TNF-α), **(C)** Granzyme B, **(D)** perforin, and **(E)** IL-17A of MAIT cells from patients with PBC (*n* = 6) and from HCs (*n* = 6). Gray plots represent fluorescence minus one (FMO) control. The data were expressed as mean ± SD. NS, not statistically significant; *p < 0.05, ***p < 0.001, and ****p < 0.0001 by Student's *t-*test.

We further investigated the immunophenotype of MAIT cells from patients with PBC. We found that the activation markers, CD69 (35.0 ± 32.0% vs. 11.4 ± 11.2%, *p* < 0.01) and CD38 (12.9 ± 16.7% vs. 1.7 ± 1.6%, *p* < 0.05), were upregulated in MAIT cells from patients with PBC compared to those from HCs (Figure 2A, Supplementary Figure 2) with the stimulation of anti-CD3 and anti-CD28 antibodies, suggesting that MAIT cells from patients with PBC were over-activated. Furthermore, MAIT cells from PBC produced significantly more IFN-γ (88.3 ± 4.2% vs. 64.2 ± 10.1%, *p* < 0.01), TNF-α (93.0 ± 1.1% vs. 80.1 ± 5.3%, *p* < 0.01), Granzyme B (89.3 ± 3.3% vs. 72.1 ± 7.0%, *p* < 0.01), and perforin (46.8 ± 6.6% vs. 34.8 ± 7.7%, *p* < 0.05), but not IL-17 (Figures 2B–E), with the stimulation of PMA and ionomycin, which indicated both pro-inflammatory and cytotoxic phenotypes. Therefore, these data suggested that MAIT cells from patients with PBC were potentially activated, produced pro-inflammatory cytokines, and might be involved in the pathological process of PBC.

## CXCL12-CXCR4 Pathway Potentially Mediates Accumulation of MAIT Cells in the Liver

**Figure 3:**
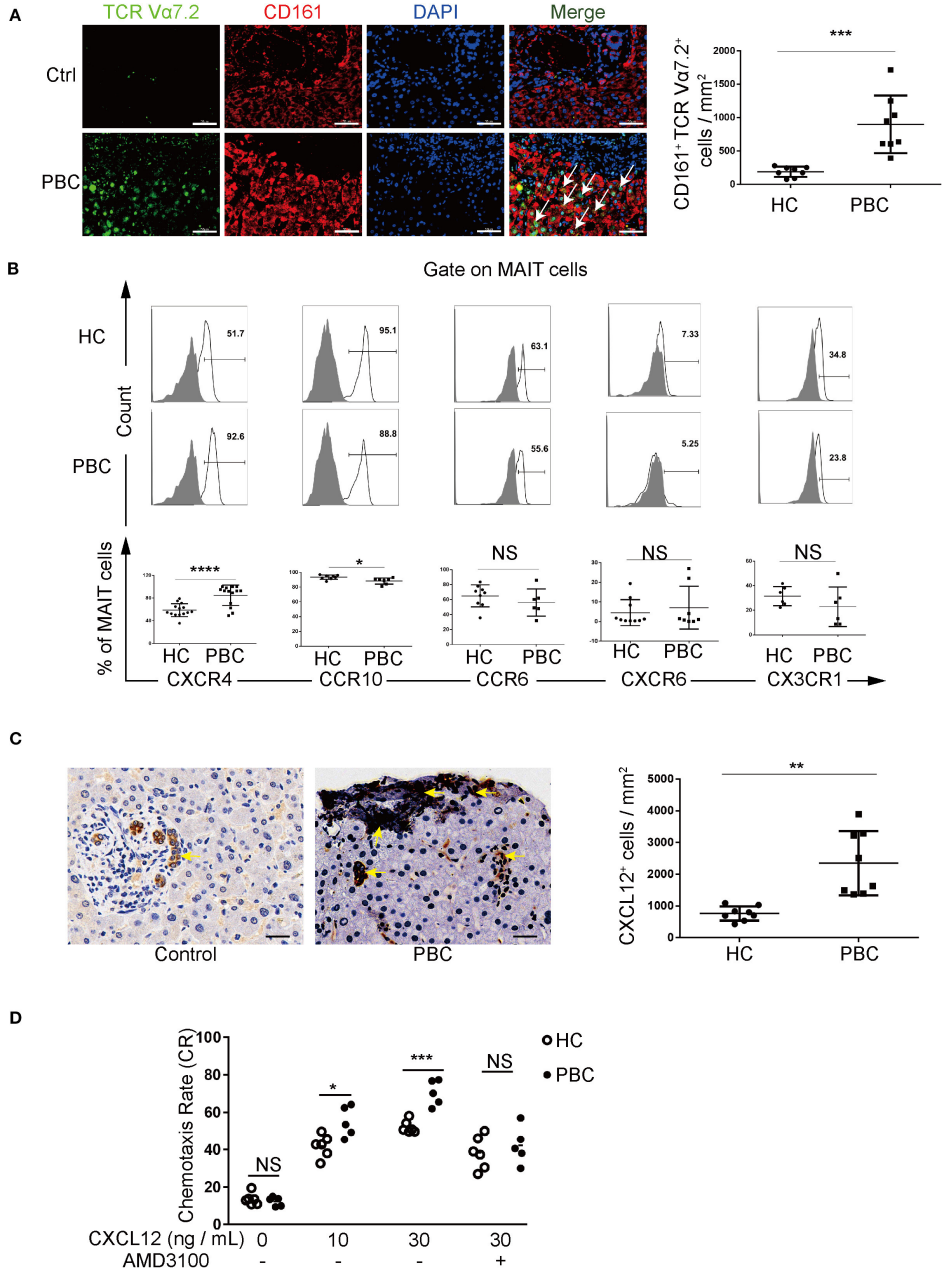
MAIT cells in patients with PBC accumulate in the liver *via* CXCL12–CXCR4 pathway. **(A)** Immunofluorescence staining of MAIT cells (arrow) in the liver of patients with PBC (*n* = 8) and in patients with hepatic hemangioma (Controls, *n* = 8). Magnification: ×400, scale bar: 50 μm. **(B)** Representative FACS plots (upper) and summary graphs (bottom) of CXCR4 (*n* = 14), CCR10, CCR6, CXCR6, and CX3CR1 on MAIT cells from patients with PBC and from HCs. **(C)** Immunohistochemistry staining of CXCL12 in the liver of patients with PBC (*n* = 8) and in Controls (*n* = 8); magnification: ×400, scale bar: 50 μm. **(D)** Chemotaxis rate of MAIT cells stimulated with CXCL12 (and) AMD3100 from patients with PBC (*n* = 5) and from HCs (*n* = 6). Gray plots represent FMO control. The data were expressed as mean ± SD. NS, not statistically significant; *p< 0.05, **p < 0.01, ***p < 0.001, and ****p < 0.0001 by the Student's *t-*test and the ANOVA analysis.

Given that the liver is the target organ for PBC and that it is enriched with MAIT cells, we analyzed the liver-resident MAIT cells using immunofluorescence staining. We found a higher number of CD161^+^TCRVα7.2^+^ MAIT cells in the liver (MAIT cells per mm^2^, 899 ± 431 vs. 189 ± 76, *p* < 0.01, Figure 3A), including the portal area (Supplementary Figure 3) from patients with PBC than those from controls. Furthermore, the apoptosis of resting (1.5 ± 1.0% vs. 1.3 ± 1.2%, *p* > 0.05) MAIT cells and activated (1.3 ± 0.5% vs. 1.5 ± 0.6%, *p* > 0.05) MAIT cells and the proliferation potential (53.1 ± 7.9% vs. 54.5 ± 6.4%, *p* > 0.05) of MAIT cells from patients with PBC were comparable to those from HCs (Supplementary Figure 4), suggesting that decreased MAIT cells might result from their accumulation in the liver.

We further explored the mechanism of accumulation of MAIT cells in the aberrant liver of patients with PBC. We screened a series of chemokine receptors expressed on MAIT cells, including CXCR4, CCR10, CCR6, CXCR6, CX3CR1 (Figure 3B), CXCR1, CXCR3, CXCR5, and CCR5 (Supplementary Figure 5). We found that MAIT cells from patients with PBC expressed higher levels of CXCR4 than those from HCs (84.8 ± 18.0% vs. 58.7 ± 11.4%, *p* < 0.01). Moreover, CXCR4 endogenous ligand CXCL12 was overexpressed in the liver of patients with PBC (Figure 3C). Furthermore, chemotaxis of MAIT cells was promoted by CXCL12 (10 ng/ml: 55.0 ± 8.2% vs. 42.0 ± 6.0%, *p* < 0.01; 30 ng/ml: 70.4 ± 6.8% vs. 52.2 ± 3.5%, *p* < 0.01, Figure 3D), which was attenuated by the CXCR4 antagonist, AMD3100 (42.3 ± 10.0% vs. 38.3 ± 8.8%, *p* > 0.05, Figure 3D). In contrast, circulating CD3^+^CD8^+^ T cells, CD3^−^CD56^+^ NK cells, and CD3^+^CD56^+^ NKT cells from patients with PBC were comparable to those from HCs (Supplementary Figure 6). Intriguingly, CD3^+^CD8^+^ T cells from PBC patients expressed higher levels of CXCR4, but these levels were lower than those in MAIT cells (50.6 ± 20.1% vs. 84.8 ± 18.0%, *p* < 0.05, Supplementary Figure 7), suggesting that CXCL12 potentially less attracted CD3^+^CD8^+^ T cells. Taken together, these data supported that MAIT cells in patients with PBC might be attracted to the liver, which was mediated by the interaction between CXCL12 and CXCR4.

### IL-18 Promotes MAIT Cell Activation

**Figure 4:**
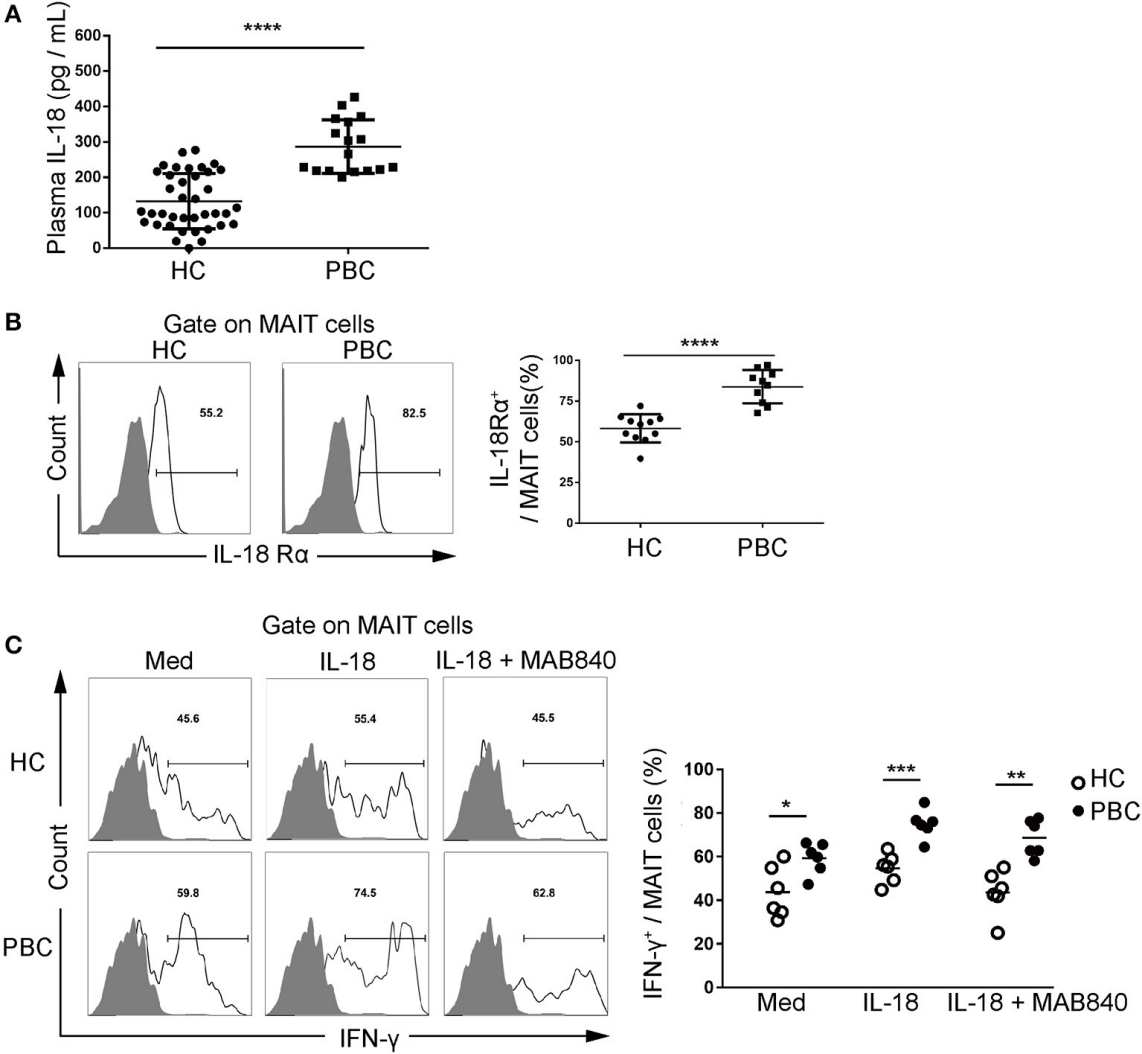
Interleukin (IL)-18 promotes MAIT cell activation. **(A)** Plasma IL-18 in patients with PBC (*n* = 17) and HCs (*n* = 38). **(B)** Representative FACS plots (left) and summary graphs (right) of IL-18Rα expression on MAIT cells from patients with PBC (*n* = 10) and from HCs (*n* = 11). **(C)** The production of IFN-γ by MAIT cells from patients with PBC (*n* = 6) and from HCs (*n* = 6), stimulated with anti-CD3 and anti-CD28, IL-18, and antagonist MAB840. Gray plots represent FMO control. The data were expressed as mean ± SD. NS, not statistically significant; *p < 0.05, **p < 0.01,***p < 0.001, and ****p < 0.0001 by the Student's *t-*test and the ANOVA analysis.

We examined the underlying mechanism of activation of MAIT cells from patients with PBC. We found that plasma IL-18 was higher in patients with PBC than in HCs (286.8 ± 75.7 pg/ml vs. 132.9 ± 78.1 pg/ml, *p* < 0.01, Figure 4A). Furthermore, MAIT cells from patients with PBC expressed higher levels of IL-18Rα than those from HCs (83.8 ± 10.2% vs. 58.3 ± 8.7%, *p* < 0.01, Figure 4B). MAIT cells from patients with PBC, stimulated with IL-18, significantly upregulated the production of IFN-γ (74.9 ± 6.6% vs. 54.7 ± 6.7%, *p* < 0.01), which was partially blocked by IL-18Rα/IL-1R5 antibody (68.6 ± 8.3% vs. 43.5 ± 4.2%, p < 0.01, Figure 4C). Thus, these data suggested that elevated circulation of IL-18 might bind to overexpressed IL-18Rα on MAIT cells and promote pro-inflammatory cytokine production in patients with PBC.

## Discussion

In this study, we confirmed that circulating MAIT cells were significantly decreased in patients with PBC. MAIT cells from patients with PBC potentially accumulated in the liver, which was promoted by CXCL12-mediated chemotaxis of CXCR4-expressing MAIT cells. Furthermore, MAIT cells from PBC patients were activated and produced pro-inflammatory cytokines,which was mediated by elevated circulating IL-18 and IL-18Rα on MAIT cells.

The present study confirm that peripheral MAIT cells have reportedly decreased in patients with PBC (15, 16). Furthermore, peripheral MAIT cells in patients with PBC were inversely correlated with serum ALP, which is a disease activity index of PBC. Although half of the patients with PBC have hyperlipidemia, no association between circulating MAIT cells and lipid profiles was observed. However, the underlying mechanism for the decreased circulation of MAIT cells remains controversial. Jiang et al. (16) have proposed an overactivation–exhaustion model. We found that MAIT cells were indeed potentially activated. Exhaustion markers, such as PD-1, CD39, and Tim-3, were expressed by liver-resident MAIT cells, which might be induced by chronic stimuli exposure. Our data showed that circulating MAIT cells expressed lower CD39 in patients with PBC than in HCs (data not shown). However, a higher apoptotic potential in MAIT cells from patients with PBC was not observed, which did not support the hypothesis that decreased peripheral MAIT cells were induced by excessive post-activation of cell death. We noted that MAIT cells in patients with PBC mainly resided in the liver, which was consistent with the findings of Jiang et al. (16). However, Setsu et al. (15) noted that MAIT cells in patients with PBC were scarce in the liver. This discrepancy may be due to different PBC stages among studies. Similarly, excessive liver infiltration of MAIT cells is reported in autoimmune hepatitis and non-alcoholic fatty liver (14, 20). Furthermore, T cells and aberrant chemotaxis of MAIT cells are implicated in rheumatoid arthritis and multiple sclerosis, respectively (21, 22). Therefore, the data from this study as well as from other studies supported the hypothesis that decreased circulating MAIT cells from patients with PBC were induced by excessive infiltration into the liver through aberrant chemotaxis.

Chemokines and chemokine receptors orchestrated chemotaxis. We screened T cell-expressing receptors, including CXCR4, CCR10, CCR6, CXCR6, CX3CR1, CXCR1, CXCR3, CXCR5, and CCR5, on MAIT cells (23). We found that CXCR4 was abnormally elevated in MAIT cells from patients with PBC. Furthermore, serum CXCL12, the CXCR4 ligand, is higher in patients with PBC (24). CXCL12 is secreted by the bile duct epithelium, especially in inflammatory conditions (25, 26). We demonstrated that CXCL12 chemoattracted MAIT cells *in vitro*. Taken together, the inflammatory milieu of the portal area in patients with PBC might promote the bile duct epithelium to produce CXCL12, which subsequently recruits CXCR4^+^MAIT cells. However, CXCR4-expressing CD3^+^CD8^+^ T cells from patients with PBC did not significantly decrease in the peripheral blood, which might attribute to lower levels in the expression of CXCR4 compared to that by MAIT cells. Thus, our study suggested that targeting the CXCL12-CXCR4 pathway might be a potential therapeutic approach to alleviate MAIT cell infiltration into the liver of patients with PBC.

Mucosal-associated invariant T cells from patients with PBC produced higher pro-inflammatory cytokines, Granzyme B, and perforin, indicating that MAIT cells were pathogenic to bile ducts. PBC is characterized by small bile duct inflammation, and TNF-α and IFN-γ produced by MAIT cells might contribute to the apoptosis of the intrahepatic bile duct (23). MAIT cells are activated by TLR ligands, such as viral DNA and lipopolysaccharide, and cytokines (27), such as IL-7, IL-12, and IL-18. Jiang et al. (16) reported that IL-7 promotes the activation of MAIT cells in patients with PBC. Serum IL-18 levels are higher in patients with PBC and are positively correlated with cirrhosis (28). Therefore, we observed that IL-18Rα on MAIT cells from patients with PBC was upregulated, and IL-18 indeed promoted the activation of MAIT cells. Taken together, we identified a new mechanism of IL-18-mediated activation of MAIT cells, which added a new layer of hyperactivation of MAIT cells in patients with PBC and suggested that IL-18 and IL-18R might be a therapeutic target for PBC.

In conclusion, MAIT cells from patients with PBC accumulated in liver *via* CXCL12-CXCR4-mediated chemotaxis, produced pro-inflammatory cytokines, and contributed to portal inflammation, which was potentially mediated by elevated IL-18. Targeting MAIT cells may be a therapeutic approach for PBC.

## Data Availability Statement

The original contributions presented in the study are included in the article/Supplementary Material, further inquiries can be directed to the corresponding author/s.

## Ethics Statement

The studies involving human participants were reviewed and approved by Institutional Review Board of PUMCH. The patients/participants provided their written informed consent to participate in this study.

## Author Contributions

FZ and HC designed and supervized the study. SL, LW, and CH collected samples. ZC and JS collected data. ZC performed the experiments and statistical analysis. ZC and HC drafted and revised the manuscript. FZ obtained funds. All authors contributed to the article and approved the submitted version.

## Conflict of Interest

The authors declare that the research was conducted in the absence of any commercial or financial relationships that could be construed as a potential conflict of interest.
