# Supplementary material for: CXCL12-CXCR4-Mediated Chemotaxis Supports Accumulation of Mucosal-Associated Invariant T Cells Into the Liver of Patients With PBC
Source: Front Immunol. 2021 Mar 19;12:578548. doi: 10.3389/fimmu.2021.578548 (PMC8017208; doi:10.3389/fimmu.2021.578548)
Supplement: Supplementary file 1 [file Data_Sheet_1.docx]

Supplementary Material

## Supplementary Figure Legends

**Fig. S1.** Correlation of peripheral MAIT cell population with laboratory parameters in PBC patients and healthy controls.

Correlation of peripheral MAIT cell population with (**A**) DBIL, (**B**) ALT, (**C**) TC, (**D**) UA, (**E**) IgG, (**F**) TG in PBC patients (n=55), with (**G**) TC and (**H**) TG in HCs (n=69). ALT, alanine aminotransferase; DBIL, direct bilirubin; IgG, immunoglobulin G; TC, total cholesterol; TG, total triglycerides; UA, uric acid. Correlations were calculated using spearman’s correlation analysis.

**Fig. S2** Mean Fluorescence Intensity (MFI) of Granzyme B, CD38, CD69, perforin, IL-18Rα, and CXCR4 on MAIT cells in PBC patients and healthy controls.

MFI of (**A**) Granzyme B (PBC=6, HC=6), (**B**) CD38 (PBC=8, HC=11), (**C**) CD69 (PBC=24, HC= 42), (**D**) perforin (PBC=6, HC=6), (**E**) IL-18Rα (PBC=10, HC=12), and (**F**) CXCR4 (PBC=14, HC=14) on MAIT cells in PBC patients and HCs, respectively. Data were expressed as mean±SD. **p*<0.05, ****p*<0.001, *****p*<0.0001 by student’s *t*-test.

**Fig. S3** Immunofluorescence staining of MAIT cells in liver portal area of a PBC patient.

Immunofluorescence staining of DAPI (blue), CD161 (red), and TCRVα7.2 (green) of MAIT cells in liver portal area of a PBC patient. Magnification: ×400, scale bar: 50 μm.

**Fig. S4** Apoptosis and proliferation of MAIT cells from PBC patients and healthy controls.

(**A**) Representative FACS plot (left) and summary graphs (right) of apoptosis of freshly isolated MAIT cells from PBC patients (n=20) and HCs (n=19). (**B**) Representative FACS plot (left) and summary graphs (right) of apoptosis of activated MAIT cells from PBC patients (n=8) and HCs (n=8) stimulated with anti-CD3 and anti-CD28 for 72 h. (**C**) Representative FACS plot (left) and summary graphs (right) of CFSE dilution of MAIT cells from PBC patients (n=8) and HCs (n=8) stimulated with anti-CD3 and anti-CD28 for 72 h. Grey plots represent FMO control. Data were expressed as mean±SD. NS, not significant by student’s *t-*test.

**Fig. S5** Chemokine receptor profiles on MAIT cells from PBC patients and healthy controls.

Representative FACS plot (upper) and summary graphs (bottom) of CXCR1 (PBC=10, HC=10), CXCR3 (PBC=18, HC=28), CXCR5 (PBC=20, HC=32), CCR5 (PBC=3, HC=3) on MAIT cells from PBC patients and HCs, respectively. Grey plots represent FMO control. Data were expressed as mean±SD. NS, not statistically significant by student’s *t-*test.

**Fig. S6** Circulating CD3^+^CD8^+^ T cells, CD3^-^CD56^+^ NK cells, and CD3^+^CD56^+^ NKT cells in PBC patients.

(**A**) Representative FACS plot (left) and summary frequency (right) of circulating CD3^+^ CD8^+^ T cells from PBC patients (n=13) and HCs (n=29). (**B**) Representative FACS plot (left) and summary frequency (right) of circulating CD3^-^CD56^+^ NK cells and CD3^+^ CD56^+^ NKT cells from PBC patients (n=16) and HCs (n=27). Data were expressed as mean±SD. NS, not statistically significant by student’s *t-*test.

**Fig. S7** Chemokine receptors on circulating CD3^+^CD8^+^ T cells from PBC patients and healthy controls.

Summary graphs of CXCR4 (PBC=9, HC=9), CCR10 (PBC=5, HC=5), CCR6 (PBC=11, HC=12), CXCR6 (PBC=5, HC=5), CX3CR1 (PBC=11, HC=7), CXCR1 (PBC=5, HC=5), CXCR3 (PBC=13, HC=11), CXCR5 (PBC=8, HC=11), and CCR5 (PBC=4, HC=5) on MAIT cells from PBC patients and HCs, respectively. Data were expressed as mean±SD. NS, not statistically significant; ***p*<0.01, *****p*<0.0001 by student’s *t-*test.

**Supplementary Figures**

**
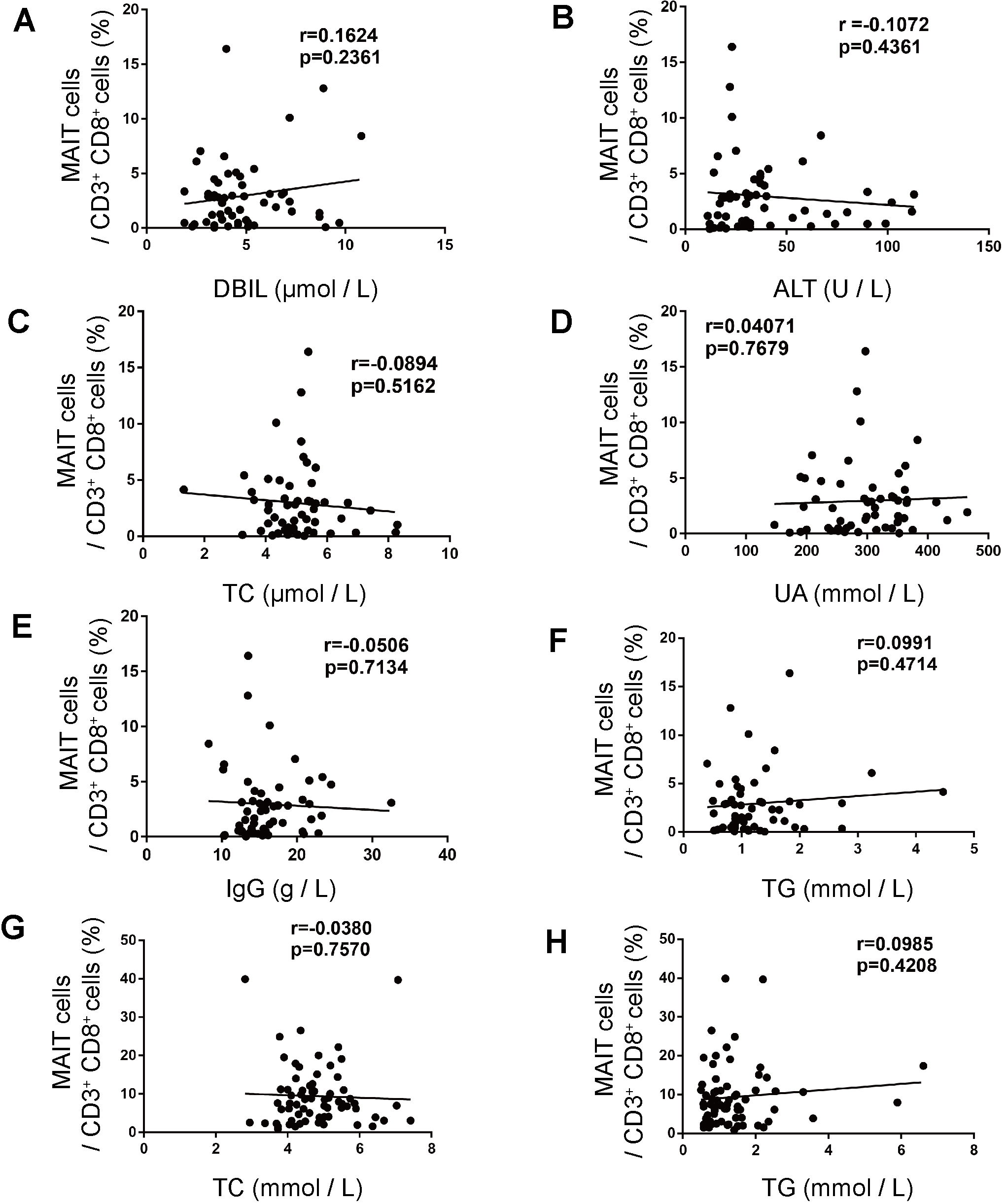
**

**Fig. S1.** Correlation of peripheral MAIT cell population with laboratory parameters in PBC patients and healthy controls.


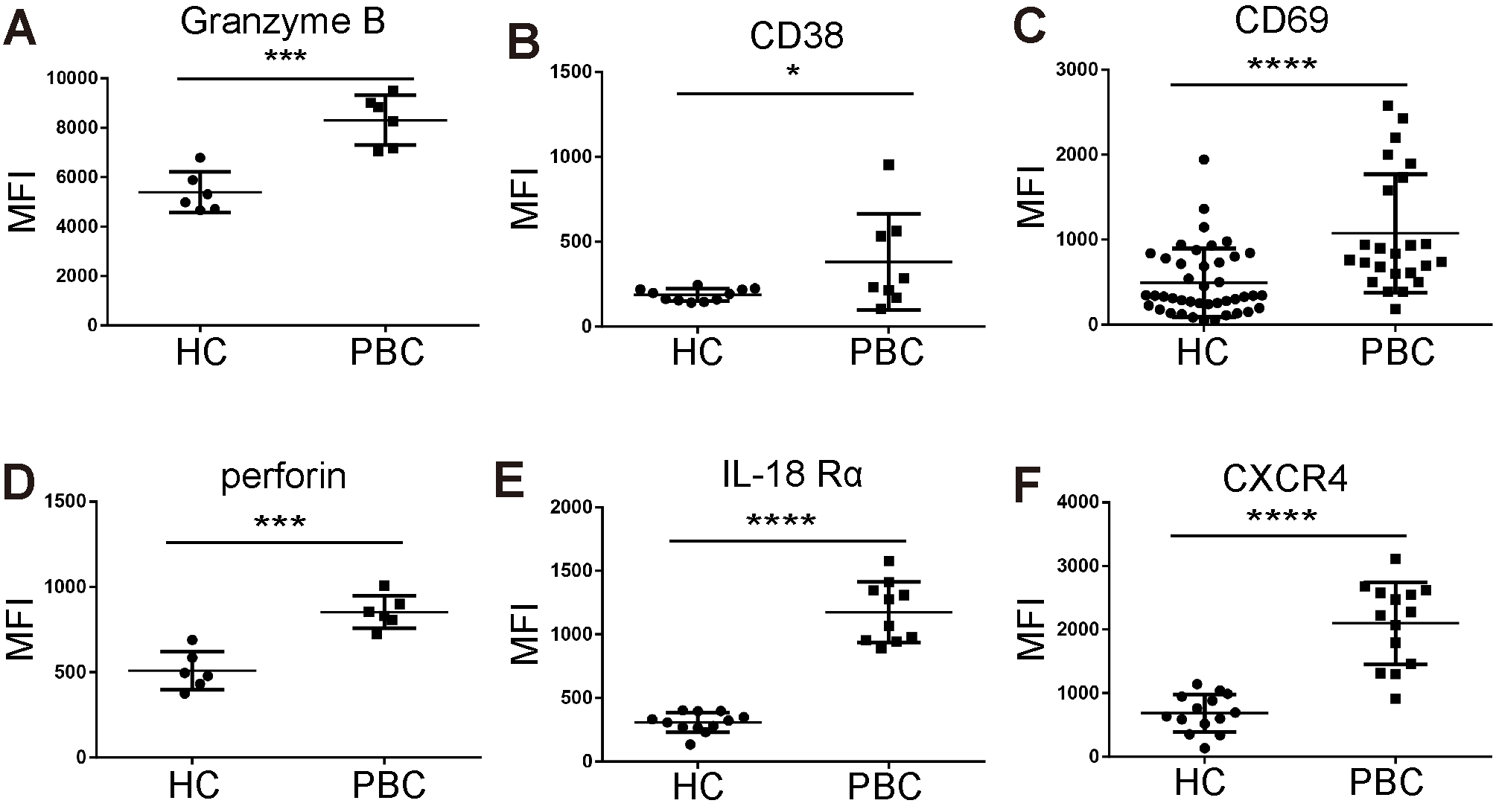


**Fig. S2** Mean Fluorescence Intensity (MFI) of Granzyme B, CD38, CD69, perforin, IL-18Rα, and CXCR4 on MAIT cells in PBC patients and healthy controls.


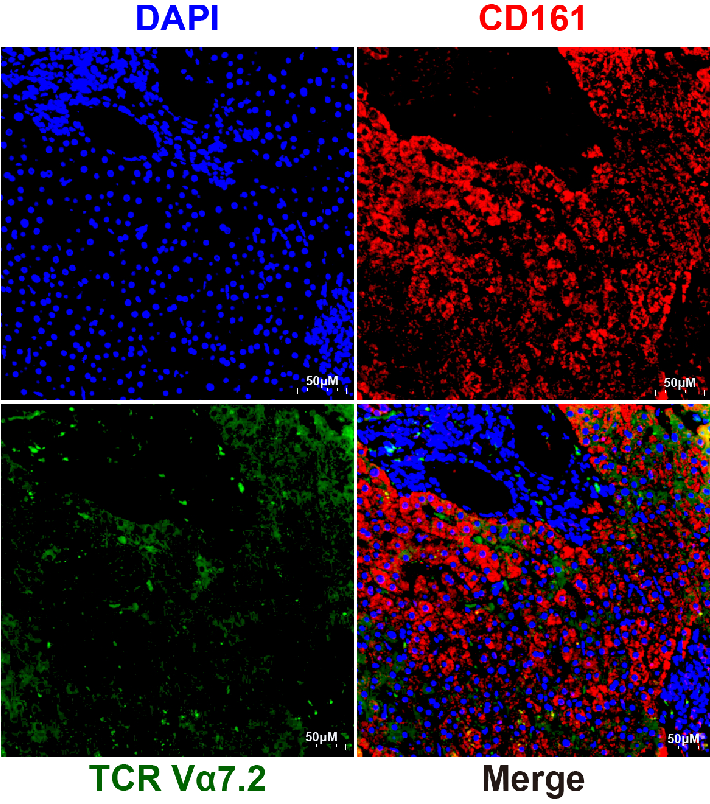


**Fig. S3** Immunofluorescence staining of MAIT cells in liver portal area of a PBC patient.


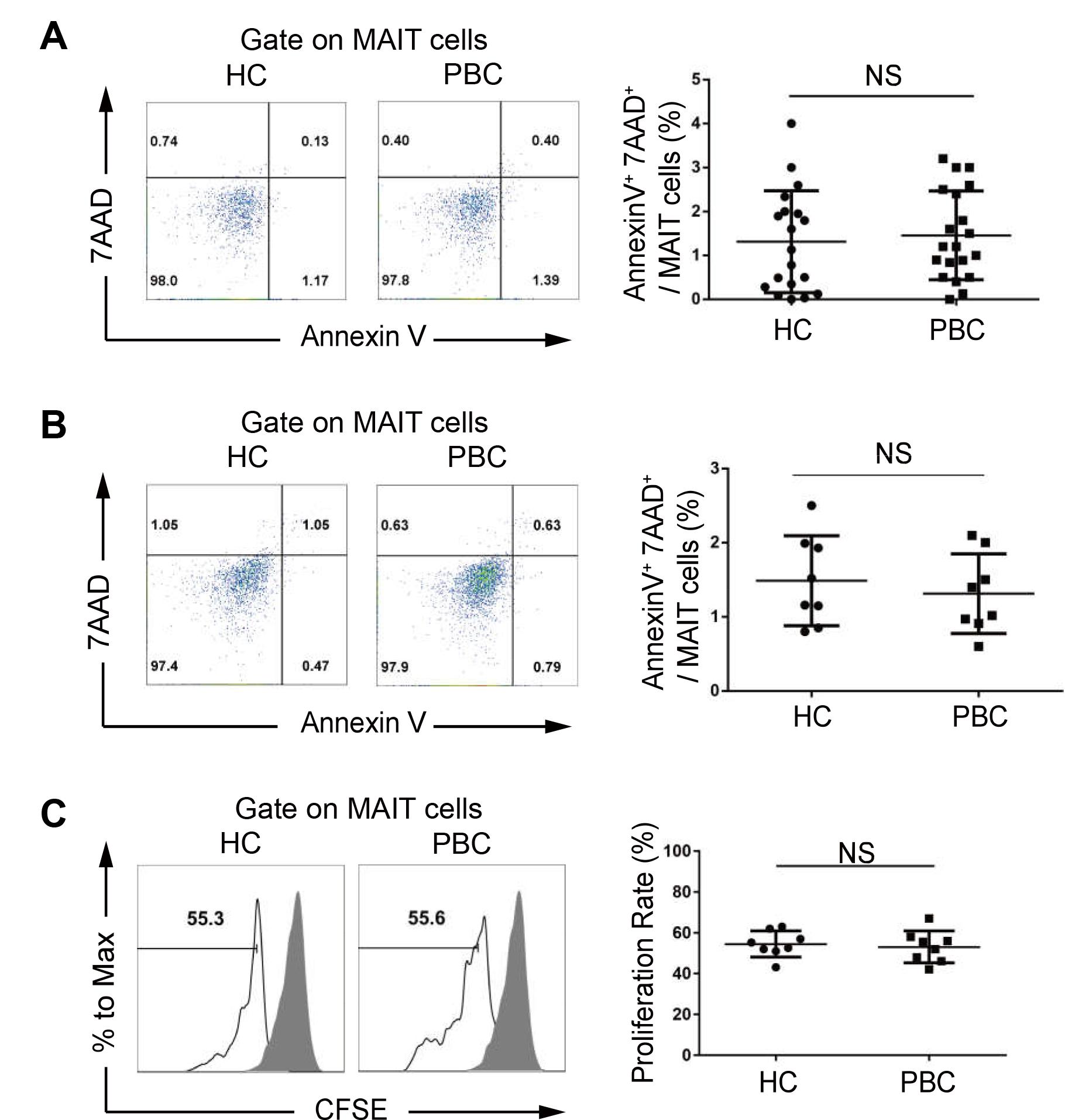


**Fig. S4** Apoptosis and proliferation of MAIT cells from PBC patients and healthy controls.


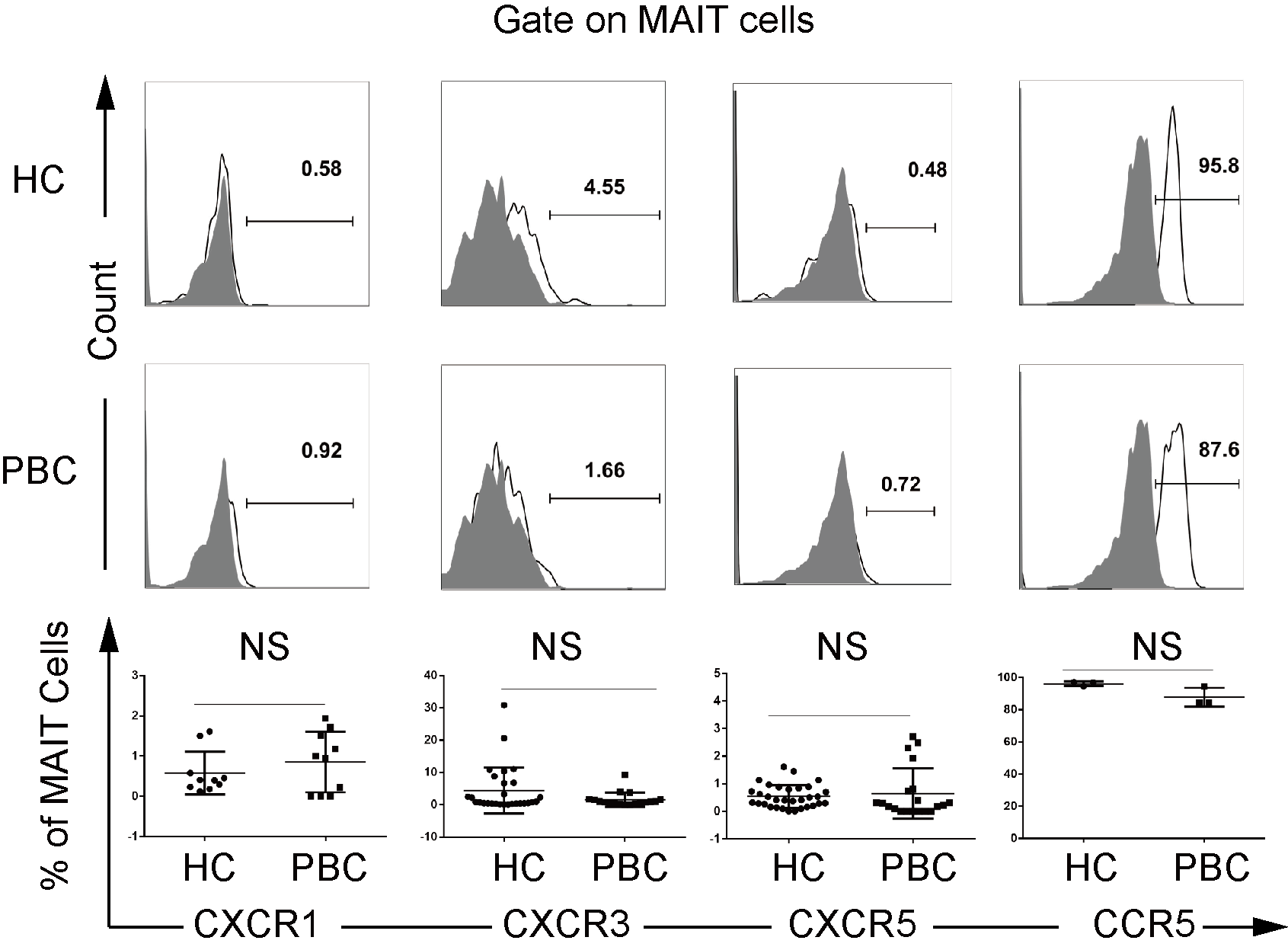


**Fig. S5** Chemokine receptor profiles on MAIT cells from PBC patients and healthy controls.


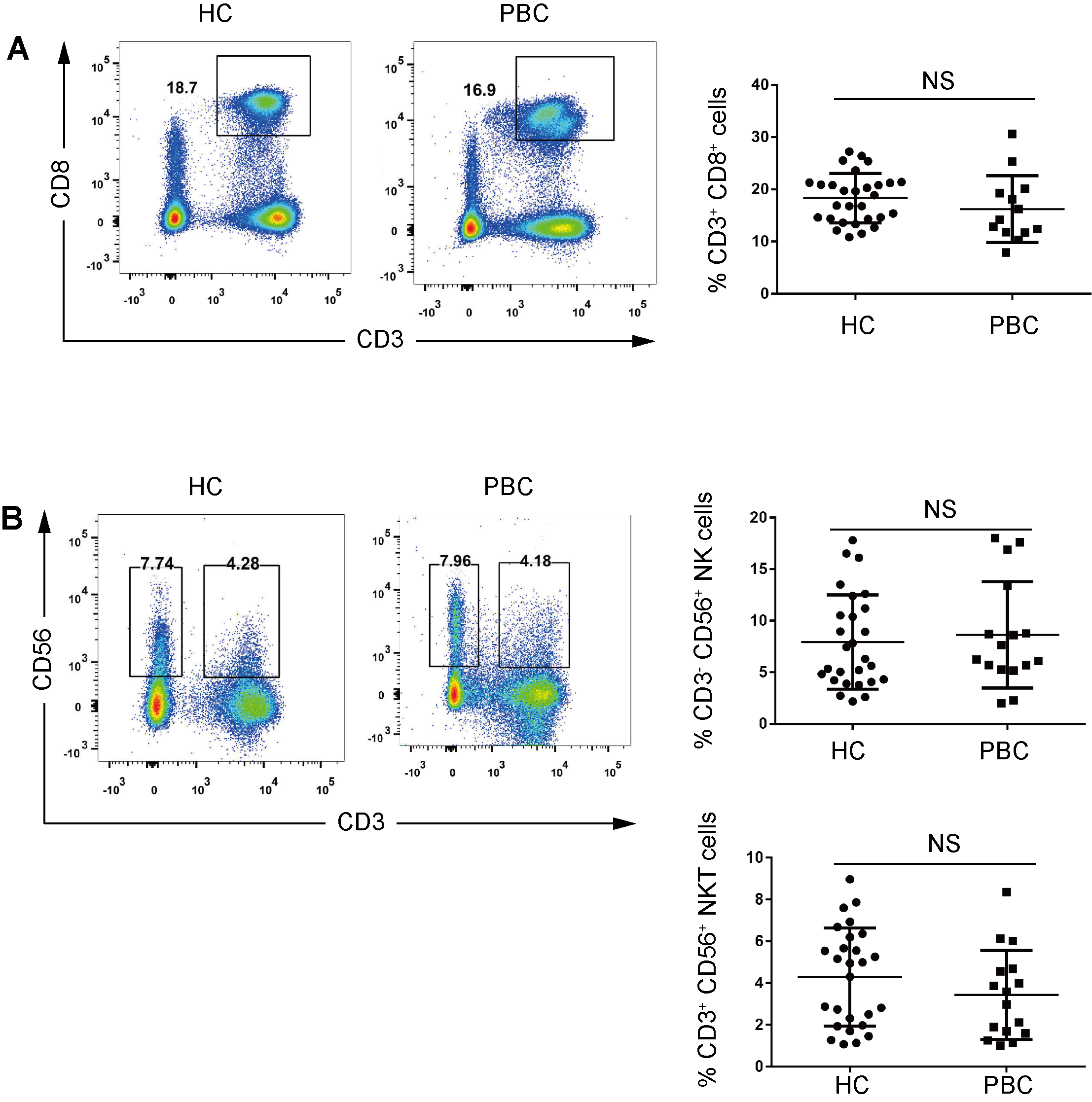


**Fig. S6** Circulating CD3^+^CD8^+^ T cells, CD3^-^CD56^+^ NK cells, and CD3^+^CD56^+^ NKT cells in PBC patients.


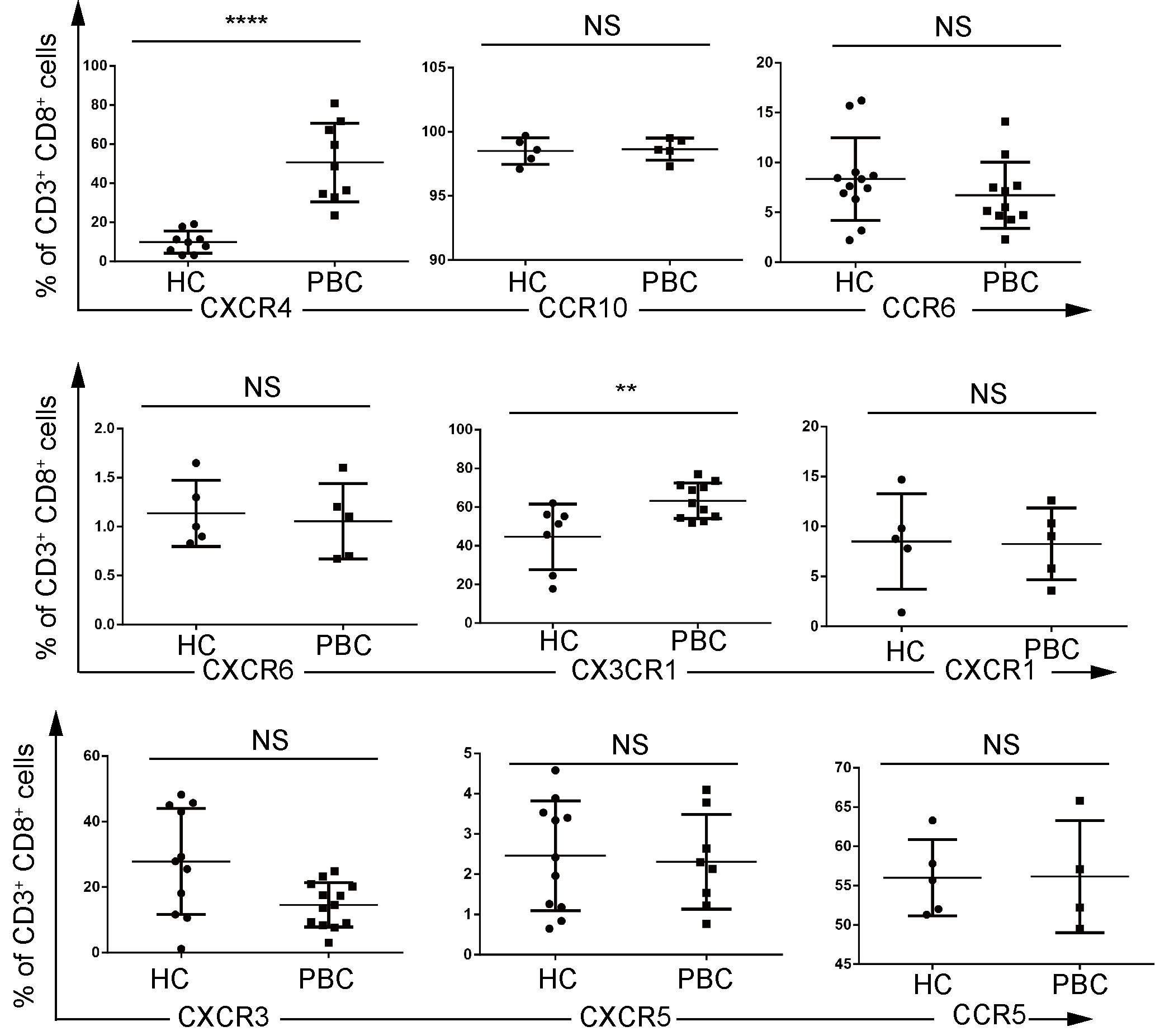


**Fig. S7** Chemokine receptors on circulating CD3^+^CD8^+^ T cells from PBC patients and healthy controls.
